# Comprehensive Transcriptomic Analysis for Developing Seeds of a Synthetic *Brassica* Hexaploid

**DOI:** 10.3390/plants9091141

**Published:** 2020-09-03

**Authors:** Zhengyi Liu, Ruihua Wang, Jianbo Wang

**Affiliations:** College of Life Sciences, Wuhan University, Wuhan 430072, China; liuzhengyi@whu.ed.cn (Z.L.); wangruihua@whu.edu.cn (R.W.)

**Keywords:** transcriptome, high-throughput sequencing, gene expression, polyploid, developing seed, *Brassica*

## Abstract

Polyploidization is a universal phenomenon in plants and plays a crucial role in evolution. In this study, the transcriptomes of developing seeds of a synthetic *Brassica* hexaploid and its parents (*B. rapa* and *B. carinata*) were analyzed to find the gene expression changes in hexaploid seeds. There were 3166 and 3893 DEGs between the *Brassica* hexaploid and its parents at the full-size stage and mature stage, respectively, most of which were upregulated in hexaploid seeds compared to its parents. At the mature stage, the hexaploid seeds showed a greater difference from its parents. These DEGs had a wide range of functions, which may account for the physiological and morphological differences between the *Brassica* hexaploid and its parents. The KEGG pathway analysis revealed that hexaploid seeds had higher levels of expression of genes involved in metabolic pathways, RNA transport and biosynthesis of secondary metabolites, and the expression levels in the photosynthesis-related pathways were significantly higher than those in *B. rapa*. Transgressive expression was the main non-additive expression pattern of the *Brassica* hexaploid. The gene expression difference between the *Brassica* hexaploid and its paternal parent was more significant than that with its maternal parent, which may be due in part to the cytoplasmic and maternal effects. Moreover, transcription factor genes, such as *G2-like*, *MYB* and *mTERF*, were highly expressed in hexaploid seeds, possibly promoting their resistance to stress. Our results may provide valuable insights into the adaptation mechanisms of polyploid plants.

## 1. Introduction

Polyploidy is a ubiquitous phenomenon in eukaryotes, resulting in important ecological and evolutionary processes. It is believed that all the angiosperms experienced at least one polyploidization process during evolution [1,2,3]. To date, more than 70% of flowering plants have been identified as polyploids [4]. According to their origin, polyploid species can be divided into autopolyploid and allopolyploid. Autopolyploidy results from the doubling of a diploid genome, while allopolyploidy is due to a combination of two or more different genomes [5,6]. Generally, the presence of larger fruits, leaves and grains in polyploid crop varieties can improve their outputs compared to those of diploids. Because polyploid species exhibit characteristics that are adaptive to various biological and abiotic stresses, such as drought, salinity, extreme temperatures and resistance to a variety of pathogenic diseases, these plants are likely to adapt to future climate change [7,8]. Many crops are cultivated as allopolyploids, including upland cotton (*Gossypium hirsutum*), oilseed rape (*Brassica napus*), and bread wheat (*Triticum aestivum*) [5].

Polyploidy leads to genetic and epigenetic changes, resulting in the reprogramming of transcriptomes, recombinant proteomes and metabolomes [1]. Genetic changes include DNA loss, homologous recombination, epistasis, ectopic recombination and gene conversion. Furthermore, the epigenetic changes, including DNA methylation, small RNA-mediated gene silencing, histone modification and transposon suppression/release, may occur at the transcriptional or posttranscriptional level [5,9]. These changes may bring about altered gene expression in the new allopolyploid [10,11]. Repeated copies of genes with similar or redundant functions during polyploid formation may alter their gene expression patterns, including unequal parental contributions, transcendental up- or down regulation and silencing [12,13]. Changes in gene expression can help overcome incompatibility triggered by allopolyploidy [14].

Transcription factors, as a kind of important regulatory factors, play an important role in the growth and development of plants. Transcription factors (TFs) also have an important impact on the adaptability of polyploids, such as influencing the morphology of polyploid rapeseed [15], the expression of non-additive genes in polyploid *Arabidopsis thaliana* [16] and the biosynthesis of GSL in polyploid *Brassica* species [17,18].

In the early years, molecular markers and microarrays were used to study the changes in genomes and transcriptomes [19,20]. Compared with these methods, high-throughput RNA sequencing (RNA-seq) can provide information on whole-genome gene expression with low background signals, more accurate quantification, a large dynamic range in expression levels and high levels of reproducibility [21]. In recent years, RNA-seq has been frequently used to investigate the transcriptomes of plant seeds, such as cotton [22], wheat [23], *Brassica napus* [24] and chrysanthemum [25].

Oilseed rape as an oil crop is widely cultivated around the world, which is essential for human nutrition. Many scholars have previously conducted in-depth transcriptomic research on oilseed rape, such as the leaf [15]. Synthetic polyploidy with known parents helps to explore the effects of polyploidization by comparing an allopolyploid and its parents. *Brassica* is normally used as a model system for tracking early genomic changes after allopolyploidization [26]. The trigenomic *Brassica* hexaploid is derived from a cross between tetraploid and diploid species and successive genome doubling [27]. Some studies have focused on the transcriptome changes at the early stages of embryonic development in rape, but few have been done on the later stage of maturation in polyploid rapeseed. We analyzed the transcriptome of seeds in a *Brassica* hexaploid (BBCCAA, 2n = 54) and its parents, *B. carinata* (BBCC, 2n = 34) and *B. rapa* (AA, 2n = 20), at two stages of late maturation by RNA-seq. In our transcriptome analysis, we studied differences in gene expression at two stages of maturation, while also paying attention to gene function, including that related to seed dormancy, chlorophyll degradation, hormones, color of the seed coat and so on. Several gene families associated with hexaploid stress resistance are also discussed in this paper. The changes in the gene expression of hexaploid seeds during two periods were of particular concern, as well as the differences between hexaploid seeds and the two parents. Therefore, this study can help us better understand the transcriptomic changes in seed development on account of polyploidization.

## 2. Results

### 2.1. Overview of Gene Expression in the Seeds of a Brassica Hexaploid and Its Parents

In this study, RNA samples were isolated from developing seeds of a synthetic *Brassica* hexaploid and its parents at the full-size and mature stage. After sequencing, an average of 6.71 Gb nucleotides was generated for each sample (a total of 120.8 Gb for all the 18 samples). After eliminating all adapter sequences, ambiguous reads, duplicate sequences and low-quality reads, 805,116,806 sequence reads were obtained from the 18 libraries of the *Brassica* hexaploid and its parents (Table 1).

**Figure 1:**
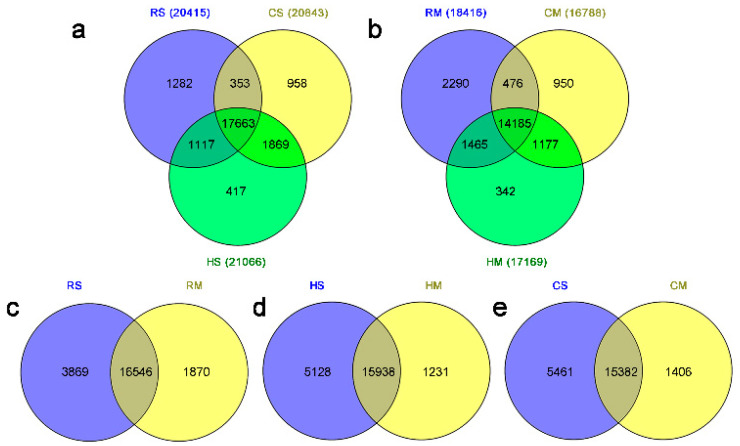
Venn diagram representing the genes expressed in the *Brassica* hexaploid and its parents at the full-size and mature stages. (**a**) Expressed genes between the *Brassica* hexaploid and its parents at the full-size stage. (**b**) Expressed genes between the *Brassica* hexaploid and its parents at the mature stage. (**c**) Expressed genes of *B. rapa* between the two stages. (**d**) Expressed genes of the *Brassica* hexaploid between the two stages. (**e**) Expressed genes of *B. carinata* between two stages. RS, *B. rapa* at the full-size stage; RM, *B. rapa* at the mature stage; HS, *Brassica* hexaploid at the full-size stage; HM, *Brassica* hexaploid at the mature stage; CS, *B. carinata* at the full-size stage; CM, *B. carinata* at the mature stage.

**Figure 2:**
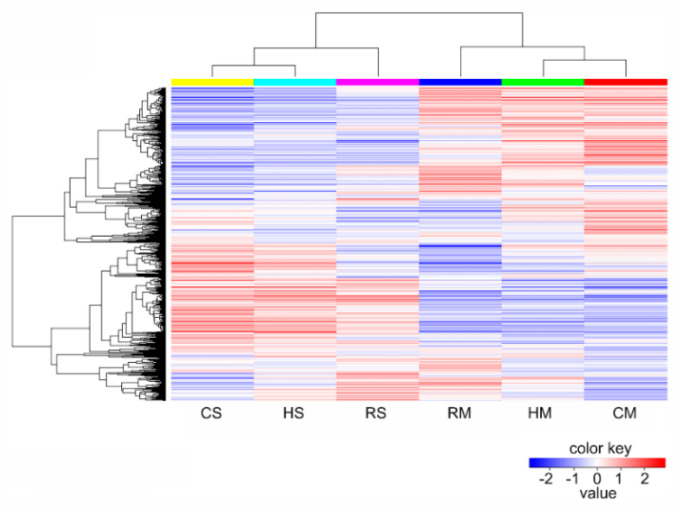
Hierarchical clustering analysis of 13,052 genes co-expressed among the *Brassica* hexaploid and its parents based on log ratio FPKM data.

**Figure 3:**
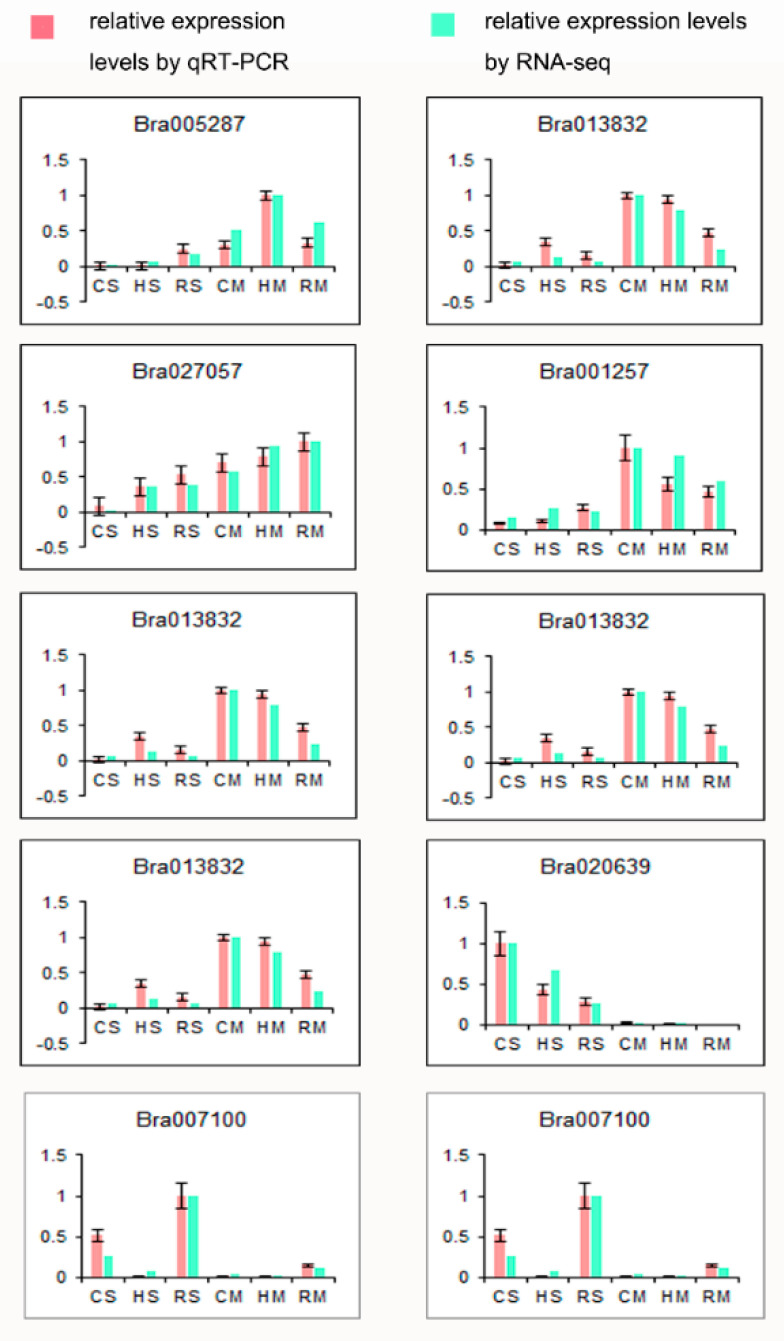
The qRT-PCR validation of the RNA-seq. Ten genes were randomly selected to verify the expression patterns in the six species. The x-axis stands for the name of the species and y-axis shows the relative expression levels. Red bars stand for the gene relative expression levels by qRT-PCR, according to the average value of each sample. Green bars represent the gene relative expression levels of the RNA-seq. In the same group, the sample with the highest expression level was regarded as “1” to normalize the gene expression level.

Using an empirical cutoff value of FPKM ≥ 1, 25,433 of 40,143 genes were detected in all the samples, 13,053 genes were co-expressed in three species at two stages and more genes were expressed at the full-size stage in all the three species. Among the 25,433 genes (ranging from 100 to ≥3,000 bp) expressed in the *Brassica* hexaploid and its parents, genes with a length of 1000–1500 bp were the richest. More genes were expressed at the full-size stage compared to the mature stage among the three species. The detailed statistical analysis of these expressed genes in the *Brassica* hexaploid and its parents at two stages is shown in Figure 1. The average ratio of each sample was 44.34%, and the uniform ratio between samples showed that the data between samples were comparable. As shown in Figure 2, hierarchical clustering was conducted on all the expressed genes in the seeds of the *Brassica* hexaploid and its parents at the two developmental stages, to explore the overall situation of the gene expression. Apparently, the number of expressed genes in the three species was significantly reduced at the mature stage compared to the full-size stage. We randomly selected ten genes in the samples to verify the expression patterns by qRT-PCR and found that the results were consistent with the results obtained by high-throughput sequencing (Figure 3).

### 2.2. Identification of the Differentially Expressed Genes (DEGs) of Seeds among the Brassica Hexaploid and Its Parents at Two Developing Stages 

**Figure 4:**
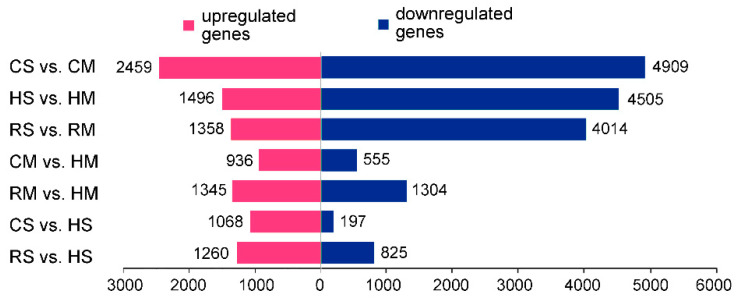
Differentially expressed genes among the *Brassica* hexaploid and its parents at two developing stages, respectively. The number of upregulated and downregulated genes of each comparison group is shown in the histogram by the magenta and blue bars, respectively.

We used the FPKM method to normalize the gene expression levels, which allows the gene expression levels to be compared within and between samples. In this study, DEGs were defined as genes with a fold change ≥ 5 and Padj (adjusted *P* value) ≤ 0.05. After screening, the number of DEGs (Figure 4) between the *Brassica* hexaploid and *B. rapa* at both stages was greater than that between the *Brassica* hexaploid and *B. carinata* (Tables S1 and S2). Among the DEGs between the *Brassica* hexaploid and its two parents, the upregulated genes accounted for the majority in the hexaploid. 

As mentioned above, at the full-size stage, among the 3166 DEGs identified in seeds between the *Brassica* hexaploid and its parent, there were 2085 DEGs between the *Brassica* hexaploid and *B. rapa* and 1265 DEGs between the *Brassica* hexaploid and *B. carinata*, respectively. By comparing the absolute foldchange of the expression levels for the 3166 DEGs, 1802 (56.9%) genes were more different in the comparison between the *Brassica* hexaploid and *B. rapa* than the comparison between the *Brassica* hexaploid and *B. carinata*, 1089 (34.7%) genes were more different between the *Brassica* hexaploid and *B. carinata* than between the *Brassica* hexaploid and *B. rapa* and there were 266 (8.4%) DEGs that showed approximately the same degree of absolute foldchange between the *Brassica* hexaploid and its two parents. Likewise, at the mature stage, among the 3893 DEGs identified between the *Brassica* hexaploid and its parent, there were 2649 DEGs between the *Brassica* hexaploid and *B. rapa* and 1491 DEGs between the *Brassica* hexaploid and *B. carinata*, respectively. By comparing the absolute foldchange of gene expression, there were 2202 (56.6%), 1331 (34.2%) and 360 (9.2%) DEGs in the same category as above. It can be seen that, at the two developing stages, the DEGs between the *Brassica* hexaploid seeds and the parents were more different from *B. rapa* in number and absolute foldchange. The overall expression of the DEGs in the *Brassica* hexaploid was more different from the paternal parents *B. rapa*. The cytoplasmic and maternal effects may explain this maternal-biased phenomenon. Cytoplasm–nuclear interactions may affect the parental genome, leading to differences in the direction and range of the genome and transcriptome changes.

**Figure 5:**
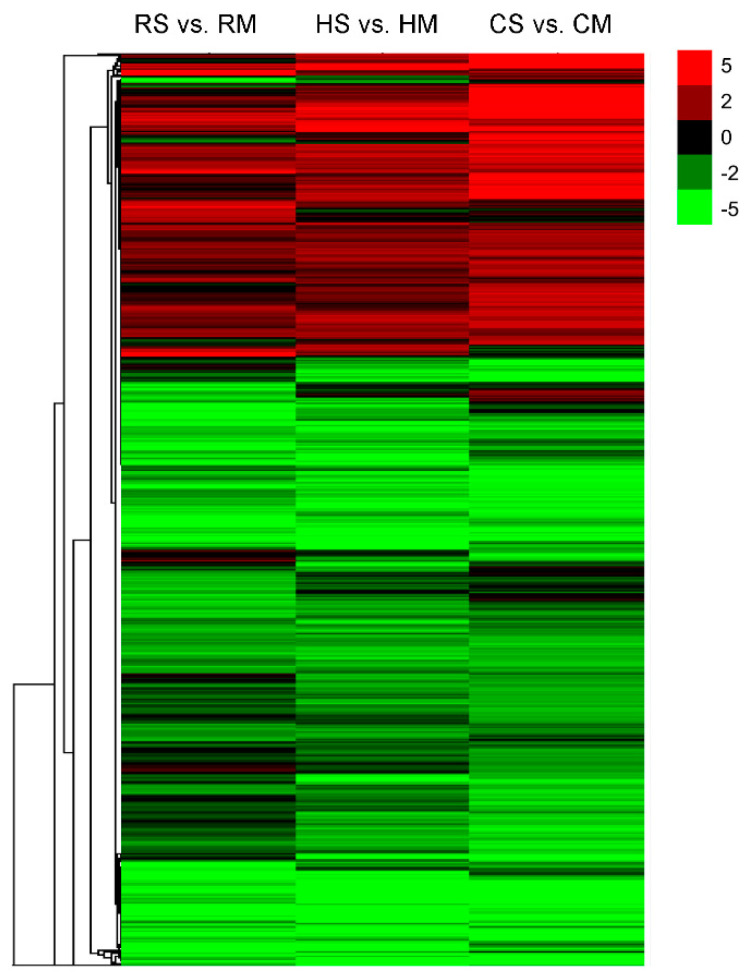
Hierarchical clustering analysis of 10,564 DEGs between two developing stages in a *Brassica* hexaploid and its parents based on log ratio FPKM data. Red represents upregulation expression and green represent downregulation at the full-size stage relative to the mature stage. Each column represents an experimental condition (e.g., HS vs. HM), and each row represents a gene.

Changes in gene expression were also observed during different developmental stages of the seeds, and most DEGs were downregulated at the mature stage compared to the full-size stage (Table S3). To classify patterns of coregulation of the DEGs during seed development in the three species, the expression profiles of these DEGs were clustered using Cluster 3.0 software. The FPKM value was transformed as the binary logarithm. As shown in Figure 5, the DEGs between the seed development stages and species were separately visualized in a different hierarchical clustering. The hierarchical clustering showed a gene expression pattern of 10,564 DEGs (Table S3) identified in the comparison of the three species at two stages. This indicated that the majority of the genes were downregulated at the mature stage, and they were extremely similar among the three species. The decrease in transcriptional activity was in line with the decline in most of the physiological activities of the seeds.

### 2.3. Functional Annotations of the DEGs of the Seeds between the Brassica Hexaploid and Its Parents at Two Stages

**Figure 6:**
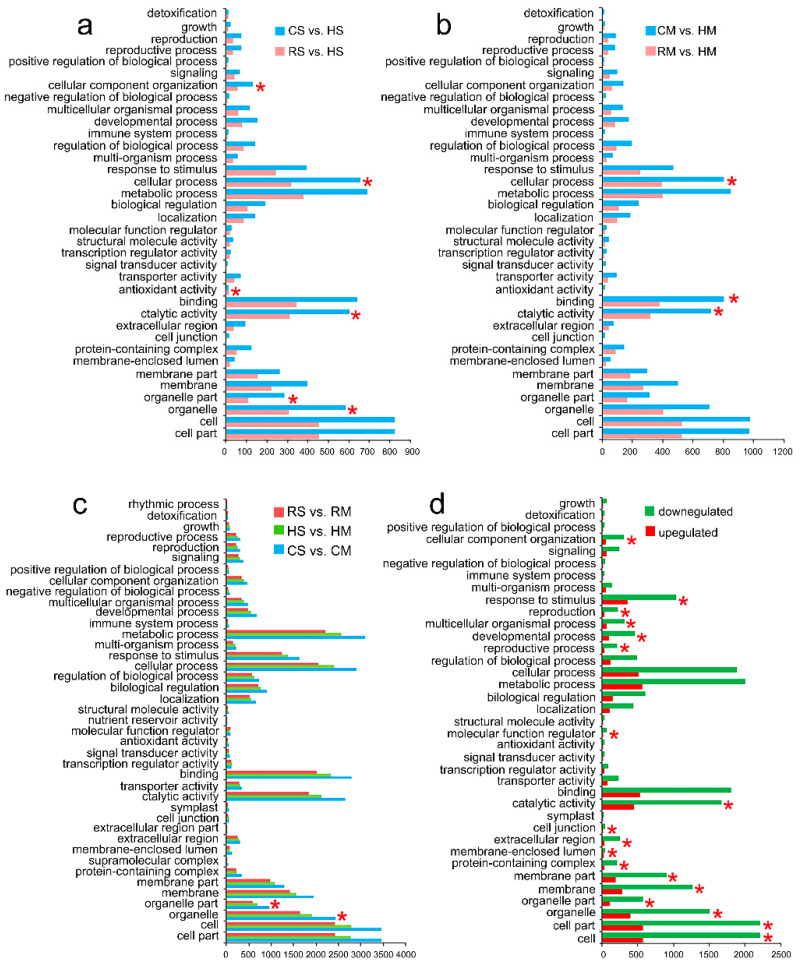
GO classifications of the DEGs. (**a**) GO classification of 3166 DEGs between the *Brassica* hexaploid and its parents at the full-size stage. (**b**) GO classification of the 3893 DEGs between the *Brassica* hexaploid and its parents at the mature stage. (**c**) GO classification of the 10,564 DEGs between the two developing stages in three species. (**d**) GO analysis of the upregulated and downregulated genes between the two stages in the *Brassica* hexaploid. An asterisk (*) stands for a statistically significant difference, with a *p* ≤ 0.05.

To analyze the functional differences of the expressed genes in the seeds, all DEGs were GO annotated and integrated according to the different comparison groups. WEGO was applied to the classify DEGs between the *Brassica* hexaploid and its parents at the full-size stage and the mature stage, respectively. A total of 10, 8 and 18 GO terms were in the category of cellular component, molecular function and biological process, respectively (Figure 6a,b). In the “cellular component” category, “cell”, “cell part” and “organelle” were the dominant terms, and in “molecular function”, “catalytic process” and “binding” were the main terms. In the “biological process” category, “metabolic process” and “cellular process” were highly represented, showing that the seeds had massive metabolic activity. In addition, “response to stimulus” was also abundant, indicating the positive protection of the seeds against biological or abiotic stress. The upregulated and downregulated DEGs between the *Brassica* hexaploid and its parents at two stages are shown in Figure S1. A total of 9, 9 and 18 terms were in the cellular component, molecule function and biological process, respectively. 

Each of the three species was compared between two successive stages as well (Figure 6c). According to the three main categories, 13, 9 and 19 terms were separately shown in the cellular component, molecular function and biological process. In the cellular component category, “cell”, “cell part”, “organelle” and “membrane” were enriched, and in the molecular function category, “catalytic process” and “binding” were dominant terms. In the biological process category, “metabolic process”, “cellular process” and “response to stimulus” were highly represented again. GO analysis of the up- and downregulated DEGs between the full-size and mature stage in the hexaploid is shown in Figure 6d. The upregulated genes account for the majority of each GO term. In the biological process category, seven out of 18 terms showed statistically significant differences between the two stages, including “cellular process”, “response to stimulus” and “reproduction”. 

To identify genes involved in the significantly enriched metabolic or signal transduction pathways, all DEGs were mapped to reference pathways in KEGG (http://www.genome.ad.jp/kegg/). At the full-size stage, between the *Brassica* hexaploid and *B. rapa*, 949 DEGs were mapped to 126 pathways, and between the *Brassica* hexaploid and *B. carinata*, 533 DEGs were mapped to 121 pathways. Based on FPKM, 76 pathways showed upregulation, but only six pathways showed downregulation in *Brassica* hexaploid seeds compared to its two parents. Among these pathways, metabolic pathways, biosynthesis of amino acids, RNA transport and ribosome exhibited considerable upregulation in the hexaploid compared to the parents. At the mature seed stage, between the *Brassica* hexaploid and *B. rapa*, 1131 DEGs were mapped to 128 pathways, and between the *Brassica* hexaploid and *B. carinata*, 605 DEGs were mapped to 121 pathways. Among these pathways, 64 pathways and seven pathways performed upregulation and downregulation in the *Brassica* hexaploid seeds compared with its two parents, respectively. RNA transport, metabolic pathways, biosynthesis of secondary metabolites, phenylpropanoid biosynthesis and galactose metabolism were sharply upregulated in the hexaploid compared to parents at the mature seed stage. According to the criteria of a Qvalue ≤ 0.05, the selected pathways were defined as significant enrichment pathways. Comparing the hexaploid with the maternal parent *B. rapa*, the main significant enrichment pathways were mismatch repair, nucleotide excision repair, DNA replication and homologous recombination. DEGs with various metabolic processes may greatly influence the physiological and morphological changes in the *Brassica* hexaploid seeds. In the comparison of the hexaploid and paternal parent *B. carinata*, pathways such as biosynthesis of secondary metabolites, flavonoid biosynthesis, phenylpropanoid biosynthesis, stilbenoid, diarylheptanoid and gingerol biosynthesis were significantly enriched. The KEGG pathways considerably altering between the *Brassica* hexaploid and its parents at two stages are summarized in Table 2. This table shows that the photosynthesis-related pathways, such as “photosynthesis—antenna proteins”, “carbon fixation in photosynthetic organisms” and “photosynthesis”, increased sharply in the hexaploid seeds relative to *B. rapa* at the full-size stage, predicting that the hexaploid may be more effective than the paternal parent in terms of photosynthesis. Moreover, Table S4 shows the KEGG pathways that considerably changed in the *Brassica* hexaploid and its parents between the two stages. Throughout these two periods, the metabolic pathways showing the most significant upregulation included other glycan degradation, biosynthesis of secondary metabolites, peroxisome, etc. Conversely, the pathways showing the most significant downregulation included RNA transport, protein export, pyrimidine metabolism and seven other pathways. 

### 2.4. Analysis of Transcription Factor (TF) Gene Expression in Seeds of the Brassica Hexaploid and Its Parents

Using HMMSEARCH [28] to align the ORF to the transcription factor protein domain (data from PlntfDB), DEGs were identified for its ability to encode TF based on the characteristics of the transcription factor family (Table S5). Most of the putative differentially expressed TF genes were downregulated in the *Brassica* hexaploid compared to *B. rapa*. Conversely, more genes were upregulated in the *Brassica* hexaploid than in *B. carinata.* Comparing the *Brassica* hexaploid with *B. rapa* and *B. carinata* at the full-size stage, 175 putative TF genes were identified, belonging to 38 families. According to FPKM, there were nine putative TF gene families that expressed the highest level in the *Brassica* hexaploid seeds at the full-size stage relative to the parents, including *ARR-B*, *bHLH*, *C2C2-Dof*, *G2-like*, *HSF*, *mTERF*, *MYB*, *Trihelix* and *ULT* gene families. In addition, the *C2C2-GATA* family had the lowest expression level in the *Brassica* hexaploid full-size seeds. At the mature stage, the comparison between the *Brassica* hexaploid and its parents identified 184 putative TF genes distributed in 40 families. The *ABI3VP1*, *C2C2-Dof*, *G2-like*, *mTERF*, *MYB* and *MYB-related* gene families showed the most abundant expression in the *Brassica* hexaploid seeds at the mature stage compared to its parents, while the *AP2-EREBP*, *bHLH* and *NAC* gene families expressed the lowest. In general, the TF gene families of *C2C2-Dof*, *G2-like*, *mTERF* and *MYB* may offer significant advantages in the late maturity of the *Brassica* hexaploid seeds compared to *B. rapa* and *B. carinata*. Among the comparisons between the full-size and mature stage of seeds in the *Brassica* hexaploid, 490 putative TF genes showed different expression, belonging to 47 TF gene families. Among the TF gene families, 24 were downregulated and 23 were upregulated based on FPKM. 

### 2.5. Non-Additive Expressed Genes in Brassica Hexaploid Seeds of Two Stages

**Figure 7:**
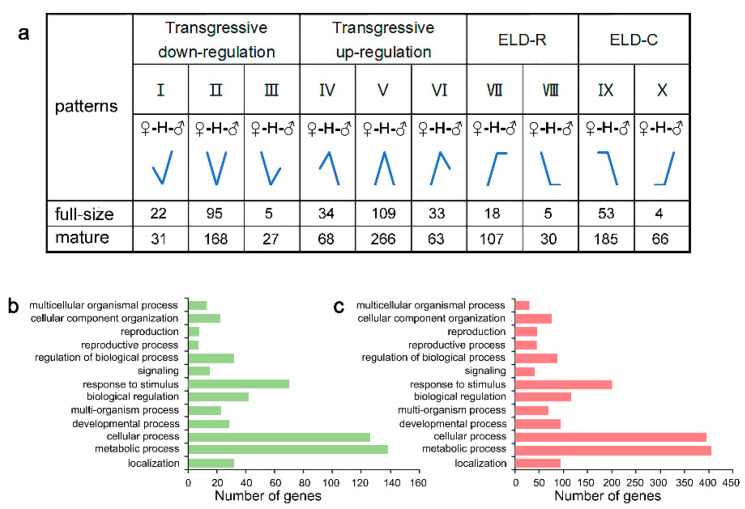
Expression patterns of non-additive *Brassica* hexaploid genes at the full-size and mature stage. (**a**) Non-additive genes between the *Brassica* hexaploid and its paternal parent *B*. *rapa* and maternal parent *B*. *carinata* at the two stages were classified into 10 patterns according to the expression levels, respectively. (**b**,**c**) Enriched GO terms relative to the “biological process” of non-additive *Brassica* hexaploid genes at the full-size stage and mature stage, respectively.

To select the non-additive expressed genes in *Brassica* hexaploid seeds, the expression levels of all the genes in hexaploid were compared with the mid-parent value (MPV). In this study, genes with a fold change ≥ 2 and Padj ≤ 0.05 were determined as non-additive genes, while the rest of the genes were additive genes (Table S6). At the full-size stage, 378 genes, accounting for 1.79% of the total genes, were determined to be non-additive in the *Brassica* hexaploid. According to the relationship between the *Brassica* hexaploid and parental expression levels, these genes were divided into ten expression patterns (Figure 7a) using the method of Yoo et al. [29]. In these patterns, “ELD” stands for expression-level dominance. Among these non-additive genes, a large number of genes appeared to be a transgressive expression (n = 298), significantly more than the parental dominance part (n = 80). Equally, 1011 genes at the mature stage, accounting for 5.89%, were non-additively expressed in the *Brassica* hexaploid. Among these non-additive genes, a total of 623 genes showed the transgressive expression pattern, and the remaining 388 genes showed the parental dominance pattern. Apparently, throughout the non-additive genes in these two periods of *Brassica* hexaploid seeds, the genes with a transgressive expression pattern appeared more extensive. GO analysis (Figure 7b,c) found that the non-additive genes at each developing stage were enriched in the process of metabolic process, cellular process and response to stimuli. 

## 3. Discussion

In recent years, extensive research has been done on allopolyploid transcriptomes, but little is known about the transcriptome of *Brassica* allopolyploid seeds at the late maturity stage. In this study, we investigated the developing seeds of a synthetic *Brassica* hexaploid and its parents by RNA-seq. We systematically analyzed the identified differentially expressed genes and predicted the adaptive alternations of gene expression in *Brassica* hexaploid seeds through functional annotation and TF gene prediction.

### 3.1. DEGs May Play an Important Role in Seed Development and Maturation in a Brassica Hexaploid 

In general, polyploids have an increase in yield compared to diploids. Furthermore, polyploids show better tolerance against stress [7]. In our study, we delved into the alternations between *Brassica* hexaploid seeds and its parents to explore the adaptive changes of polyploids at the transcriptome level. Photosynthesis is the basis of crop yield formation as more than 90% of the dry matter in crops is directly derived from photosynthesis [30]. As mentioned above, the expression level of photosynthesis-related pathways was significantly higher in the hexaploid than in *B. rapa*. Among these DEGs, 62 of 67 genes showed upregulation in the *Brassica* hexaploid relative to *B. rapa,* suggesting that the hexaploid might be more effective in photosynthesis than the paternal parent. In the comparison between the *Brassica* hexaploid and *B. rapa*, all 34 genes classified as photosynthesis-related processes in GO showed higher expression in the hexaploid. This result suggests that photosynthesis may play an important role in the process of polyploidization. The gene expression levels in the metabolic pathways and biosynthesis of secondary metabolites were significantly higher in hexaploid seeds compared to the parents, suggesting that they may have advantages in metabolic activity. 

In the later stages of embryogenesis, the seeds enter a dormant phase with dehydration, decomposition of photosynthetic organs and chlorophyll degradation [31]. The changes in RNA transport, protein export, pyrimidine metabolism and other metabolic pathways in hexaploid seed maturation were similar to the maturation process of the parent seeds. The expression of these genes was significantly reduced, indicating that the transcriptional activity was significantly reduced in the later stage of seed maturation, which was consistent with the decrease in the number of genes expressed at the mature stage. Three DEGs involved in “postembryonic development” (GO: 0009791) were abundantly expressed at the mature stage. *Bra009229* encodes an 18 kDa seed maturation protein, *Bra004981* encodes a late seed maturation protein P8B6 and *Bra000173* encodes a late embryogenesis-abundant protein. Several genes were detected in the “embryo development end in seed dormancy” (GO: 0009793) during the maturation of the hexaploid seeds. Among them, *Bra011036* showed a high level of expression during the early and mature stages of maturation, while other genes, such as *Bra040894*, *Bra033375* and *Bra004642*, were significantly upregulated at the mature stage. All of these DEGs also played a role in “response to abscisic acid” (GO: 0009737). *Bra009112*, *Bra006460*, *Bra005113* and *Bra034159* were predicted to encode a dehydration-responsive, element-binding protein, which may play roles in the dehydration process at later maturity of hexaploid seeds. Chlorophyll (Chl) degrades rapidly during the late maturity of the seed, resulting in seed-free Chl [32], an important reporter for seed maturation. Many studies proved that the NYEs-mediated chlorophyll degradation plays an important role in seed maturation and seed germination [33,34]. We found two important putative genes, *Bra019346* and *Bra013656*, which may be essential for the degradation of Chl in our materials. These two genes are homologous to NYE1 in *Arabidopsis thaliana*, indicating a distinct upregulation during the morphological maturity of the *Brassica* hexaploid and its parents. *Bra013656* received a GO annotation of “plastid” (GO:0009536) and “porphyrin-containing compound catabolic process” (GO:0006787); *Bra019346* also received the “plastid” (GO:0009536) annotation and was predicted as “protein STAY-GREEN 1, chloroplastic” in the NR annotation. It is possible that they are critical for the Chl degradation in the *Brassica* hexaploid, indicating that seeds may remain in chlorophyll degradation continuously during maturation. 

The raffinose family oligosaccharides (RFO) are one of the important indicators for the beginning of late maturation in seeds [35]. The regulation of RFO accumulation is regulated by the transcription of abscisic acid (ABA) and gibberellic acid (GA). In our study, *Bra013248* (*ABI3*), *Bra005287* and *Bra017251* (*ABI5*) in the *Brassica* hexaploid and its parents showed high expression levels. The ABI5 was upregulated at the mature stage compared to the full-size stage, consistently with the expression patterns of genes involved in oligosaccharide biosynthesis, indicating that ABA may play an important role in RFO accumulation during seed maturation. The accumulation of late embryogenesis abundant proteins (LEA protein) is also an important feature of seed maturation, which can improve plant resistance, especially dehydration resistance [36]. The synthesis of the LEA protein is induced by seed maturation signals, drought and salt stress, as well as ABA signaling. In the *Brassica* hexaploid and its parents, most of the predicted genes associated with the LEA protein were expressed in high abundance and were upregulated at the mature stage, in accordance with the expression of the ABA-related genes.

### 3.2. Putative Transcriptome Factor Genes May Promote Better Allohexaploid Adaptation

As an important regulator, transcription factors play an important role in plant growth and development. A transcriptome study using *Brassica* hexaploid leaves as materials found that the TCP and ARF gene families may have important effects on the morphology of the polyploids [15]. Transcription factors may also influence the non-additive gene expression in synthesized allotetraploid *Arabidopsis* [16]. As mentioned above, several putative TF gene families were significantly altered in the hexaploid seeds, indicating that they might be important for the development of hexaploid seeds.

The TF gene families (*G2-like*, *MYB* and *mTERF*) with the highest expression level in the hexaploid seeds are worth noticing. The *G2-like* gene family regulates chloroplast development in a variety of plant species. Studies have found that a pair of *G2-like* genes regulates chloroplast development in *Arabidopsis* [37]. The change in the expression level of a putative *G2-like* gene family in the *Brassica* hexaploid may affect the chloroplast development of its seeds. The MYB, ubiquitous in plants, is a transcription factor related to the regulation of plant growth and development, physiological metabolism, cell morphology and pattern building. For example, *MYB* transcription factors act as positive regulators in disease resistance, activating disease-resistant defense responses or regulating programmed cell death in plants, thereby increasing plant resistance [38]. In addition, two TT2-type *MYB* transcription factors were identified to be involved in the regulation of proanthocyanidin biosynthesis in a tetraploid cotton (*Gossypium hirsutum*) [39]. In *Brassica* hexaploid seeds, the putative *MYB* gene families had a higher level of expression compared to its parents at both stages. An upregulated DEG *Bra039763* between the hexaploid and *B. rapa* at the full-size seed stage was also involved in an anthocyanin-containing compound biosynthetic process by the GO annotation. Other genes, such as *Bra039763* and *Bra021708*, were related to plant hormone signaling. Among these putative *MYB* genes, *Bra002042* was upregulated in the hexaploid compared with *B. rapa* at the full-size and mature stages. Conversely, *Bra007371* and *Bra001378* were downregulated. Similarly, eight putative TF genes, such as *Bra16893*, *Bra041096* and *Bra001377*, were upregulated in the hexaploid seeds compared to *B. carinata* at both stages. These results indicate that *Brassica* hexaploid seeds might have different directions of transcriptomic changes compared to its two parents. The *mTERF* gene family is localized to the mitochondria or chloroplasts in plants, affecting developmental morphology or stress tolerance in plant [40]. This family has been confirmed to affect the abiotic stress response and regulate chloroplast function in *Arabidopsis* [41,42]. In hexaploid seeds, all six identified putative *mTERF* genes showed higher levels of expression than the parents at both stages, suggesting that they may have an important role in the seed development of the hexaploid. 

Other TF gene families that showed increased expression compared to the paternal or maternal parents were also worth exploring. The *bHLH* transcription factor is a large group of plant transcription factor families, which plays an important role in plant physiological metabolism, growth and development and stress response [43]. *Bra028443*, upregulated in the hexaploid compared with *B. rapa,* was involved in the GO annotation of “response to brassinosteroid”, “cell morphogenesis”, “regulation of timing of meristematic phase transition”, etc. Similarly, *Bra029088* was related to “postembryonic development”. The *C2C2-Dof* gene family is also one of the most important transcriptional regulators in higher plants and is involved in plant growth, development and response to abiotic stress. Its important role in abiotic stress response was determined in Chinese cabbage [44]. These putative *C2C2-Dof* genes showed a greater expression compared to that of the maternal parent *B. carinata*. Heat shock transcription factor (*HSF*) activates the heat stress response through the specific binding of organisms to heat shock elements (HSEs) under heat stress and other stress conditions, initiating the expression of downstream HSP and inducing heat shock [45]. Several identified putative TF genes showed “response to stimulus” on the GO annotation: *Bra007739* and *Bra032023* were upregulated in the hexaploid compared to *B. rapa*, and *Bra016998* was upregulated in the hexaploid compared to *B. carinata*. Increased expression of the *HSF* gene family may also contribute to the advancement of polyploid stress resistance. The *WRKY* gene family is a unique transcription factor in plants. Studies in *Arabidopsis* showed that this family is widely involved in the development and aging of plant organs, response to biological and abiotic stresses, as well as a series of physiological activities [46]. Moreover, the expression pattern of the *WRKY* gene families in *B. napus* showed substantial changes under stress conditions, also indicating that the *WRKY* genes are important for response to an environmental stress stimulus [47]. The expression level of the *WRKY* family genes in the *Brassica* hexaploid decreased relative to *B. rapa* but increased relative to *B. carinata* at both stages, suggesting that this family is more inclined to have an additive pattern in the hexaploid compared to its parents. These gene families may enhance the resistance of the hexaploid seeds and play an important role in the seed development and adaptation of the hexaploid.

### 3.3. Differential Expression of Some Genes Associated with the Synthesis of Substances Such as Flavonoids May Affect the Seed Coat Color of Mature Seeds

We found that the coat color of the *Brassica* hexaploid mature seed is closer to the *B. carinata* and distinctly darker than the *B*. *rapa* seed. Among them, the paternal parent shows yellow, while the hexaploid and its maternal parent are brown. Proanthocyanidins (PAs) are the main flavonoids that affect the color of the seed coat of *Brassica* species, which are oligomeric and polymeric end-products synthesized via flavonoid biosynthesis pathways [48,49]. In the flavonoid biosynthetic pathway, genes such as *CHI*, *CHS* and *F3H* participate in the production of a common precursor (dihydroflavonol) and are named early biosynthetic genes (EBGs) [43]. In addition, the downstream genes of this pathway (*DFR*, *LDOX* and *ANR*) are often named late-stage biosynthetic genes (*LBG*) [50]. Most of the related genes have been detected as affecting seed coat pigmentation. In the Brassicaceae, PAs accumulate in the endodermis of the seed coat, resulting in the formation of brown seed coats [51]. The expression levels of these genes related to flavonoids in *B*. *rapa* at the full-size stage was greater than that in the *Brassica* hexaploid. While expression of these genes shows a higher level in the *Brassica* hexaploid than in *B*. *rapa*, it means the expression of flavonoid-related genes has undergone great changes during the later stages of maturation, and this difference in expression at the later stages may have caused a difference in the color of the seed coat.

Previous studies have shown that different rapeseed may have their own special gene control seed coat color, such as *TT10* (*TRANSPARENT TESTA10*) [52] and *BnaC.TT2* [53] in *B. napus*, *BrTT8* [54] and *BrTT1* [55] in *B. rapa* as well as the *TT8* genes [56] and *Bra036828* [57] in *B. juncea*. Of them, *Bra036828* may be the only flavonoid biosynthetic gene and the rest are regulatory genes; this indicates that the color of the seed coat is mainly controlled by regulatory genes instead of structural genes. In our study, we found that the expression of *Bra036828* in the *Brassica* hexaploid was lower than the two parents in the two periods, and perhaps this was due to the above reason. We found the homologue gene *Bra020720* of *TT10*, *Bra025512* and *Bra028491* of *TT2* was significantly higher in the hexaploid than its two parents, which may be one of the reasons for the deeper color of the seed coat of the hexaploid seed.

## 4. Materials and Methods 

### 4.1. Plant Materials

**Figure 8:**
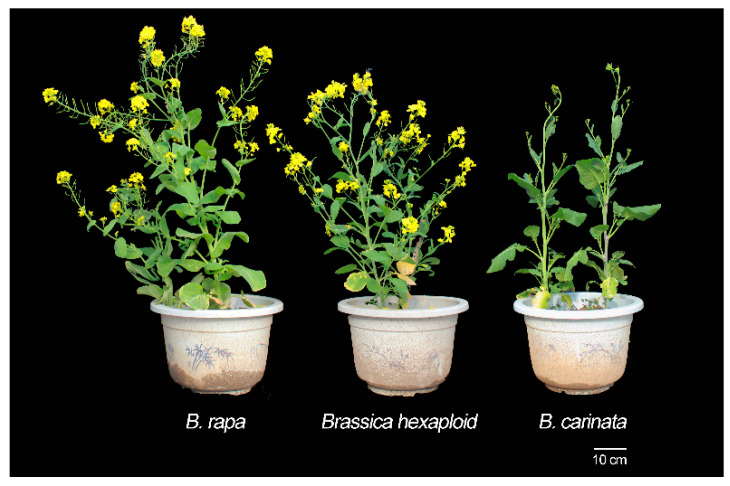
Morphology of the *Brassica* hexaploid and its parent grown for 6 months.

**Figure 9:**
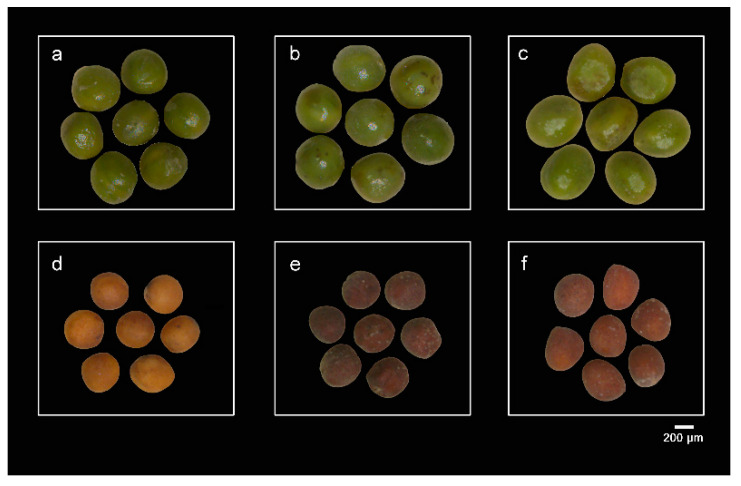
Seed morphology at two developing stages: (**a**) *B. rapa*, (**b**) *Brassica* hexaploid and (**c**) *B. carinata* at the full-size stage; (**d**) *B. rapa*, (**e**) *Brassica* hexaploid and (**f**) *B. carinata* at the mature stage. Bar represents 2 mm.

The three plant materials, namely, the tenth generation synthesized *Brassica* hexaploid (BBCCAA, 2n = 54), its paternal parent *B*. *rapa* (cultivar name, BaiguotianYC, AA, 2n = 20) and its maternal parent *B*. *carinata* (CGN03955, BBCC, 2n = 34), were planted in the greenhouse of Wuhan University, China. The *Brassica* hexaploid used in this study was described by Tian et al. (2010) [27]. Figure 8 shows the morphology of the *Brassica* hexaploid and its parent grown for 6 months. Flowers were bagged before blossoming and pollinated artificially on the first day postanthesis. Their filled seeds were collected at two developmental stages, the full-size stage (40 day after pollination) and mature stage (55 day after pollination), and each sample containing 50 seeds was taken from one plant. Three biological replicates were taken from each stage of the three materials. Seeds (Figure 9) were flash frozen and stored at −80°C for RNA extraction. 

### 4.2. RNA Extraction, cDNA Library Construction and Illumina Sequencing

Total RNAs were extracted from two-staged seeds of the three materials using TRIzol reagent (Invitrogen, Burlington, ON, Canada) according to the manufacturer’s protocol and treated with RNase-free DNase Ⅰ (Fermentas, Burlington, Canada). Purified RNAs were used to construct the RNA-seq library. In this study, the RNA sequencing library was sequenced by Illumina HiSeq^TM^ 4000. Adapter sequences and low-quality sequences were filtered from the raw reads by SOAPnuke, and clean reads were mapped to the *B*. *rapa* genome v1.5 sequences (http://brassicadb.org/brad/datasets/pub/BrassicaceaeGenome/Brassica_rapa/Bra_Chromosome_V1.5/) using HISAT (http://www.ccb.jhu.edu/software/hisat). After alignment with the reference genome, CPC software was used to predict the potential of coding for new genes.

### 4.3. Normalized Expression Levels of Genes and Gene Annotation

For gene expression analysis, RSEM (http://deweylab.biostat.wisc.edu/RSEM) was used to calculate the quantity of gene expression. The expression level of a gene was normalized by the FPKM (fragments per kilobase per million mapped fragments). The GO (gene ontology) and KEGG annotation was performed by the Blast2GO [58] and KEGG database [59], respectively. WEGO 2.0 [60] was used for GO functional classification as well as for plotting the distribution of the functions. In addition, iDEP.85 was used (http://bioinformatics.sdstate.edu/idep/) to generate a hierarchical clustering for the co-expressed genes of the *Brassica* hexaploid and its parents seeds.

### 4.4. Analysis of Differentially Expressed Genes (DEGs)

The R package DEGseq was used to determine the DEGs. The DEseq2 algorithm for differential gene detection is based on the negative binomial distribution principle [61]. Genes with a fold change ≥ 5 and Padj (adjusted *P* value) ≤ 0.05 were defined as DEGs. GO and KEGG terms were determined for DEGs. To classify DEGs with similar patterns, we used Cluster 3.0 software and Java TreeView with Pearson correlations to generate a hierarchical clustering.

### 4.5. Quantitative Real-Time PCR

To verify the accuracy of the high-throughput sequencing, ten genes (*Bra005287*, *Bra013832*, *Bra007100*, *Bra027057*, *Bra001257*, *Bra008589*, *Bra010283*, *Bra013872*, *Bra020639* and *Bra019774*) were randomly selected for qRT-PCR. The primer sequences were designed by Primer5 software and are listed in Table S7. The qRT-PCR was performed on an ABI StepOne™ Real-Time PCR System (Applied Biosystems) using SYBR Green I as a fluorescent detection dye. *ACT2/7* was used as the internal reference control to normalize the results. All reactions were performed using one biological sample with three technical replicates. The accuracy of the RNA-Seq was evaluated by comparing the relative expression of genes by qRT-PCR.

## 5. Conclusions

In this study, we analyzed the gene expression differences between the newly synthesized *Brassica* hexaploid and its parents, and found that the gene of the hexaploid seeds has undergone a wide range of changes in expression patterns relative to the parents. DEGs are involved in a wide range of biological functions, which may contribute to the environmental adaptability of the hexaploid. The analysis of the KEGG pathway and TF genes show that hexaploid may have acquired a better ability of substance metabolism synthesis and resistance. Some important genes that may be related to the late maturation of the hexaploid and its parents’ seeds were predicted and analyzed. In the study, a large amount of information was generated. On this basis, important functional or pathway-related genes can be selected for study, and phenotypic monitoring, such as plant hormones, are worthy of further discussion. Our findings may provide a new perspective for polyploid evolution mechanisms. 

## Figures and Tables

**Table 1:** Statistics of the RNA-seq (RNA sequencing) reads of developing seeds of the *Brassica* hexaploid and its parents. RS, *B. rapa* at the full-size stage; RM, *B. rapa* at the mature stage; HS, *Brassica* hexaploid at the full-size stage; HM, *Brassica* hexaploid at the mature stage; CS, *B. carinata* at the full-size stage; CM, *B. carinata* at the mature stage.

Species	Sample	Stage	Clean reads	Clean reads (%)
*B. rapa*	RS1	Full-size	44,199,458	79.60
	RS2	Full-size	44,227,522	82.06
	RS3	Full-size	44,335,290	82.26
	RM1	Mature	44,399,710	82.38
	RM2	Mature	45,242,772	81.48
	RM3	Mature	45,174,524	83.82
*Brassica* hexaploid	HS1	Full-size	45,063,192	78.83
	HS2	Full-size	44,846,972	83.21
	HS3	Full-size	44,429,210	80.01
	HM1	Mature	44,641,026	88.17
	HM2	Mature	45,300,322	89.47
	HM3	Mature	44,793,850	88.47
*B*. *carinata*	CS1	Full-size	44,761,646	80.61
	CS2	Full-size	45,066,350	83.62
	CS3	Full-size	44,490,496	80.12
	CM1	Mature	44,273,224	87.44
	CM2	Mature	44,552,900	88.00
	CM3	Mature	45,318,352	86.71

**Table 2:** The considerably changed KEGG pathways between the *Brassica* hexaploid and its parents at the two stages based on FPKM (numbers in brackets). The number before the parentheses represents the rank of the comparison group in all the comparison groups.

KEGG pathway	RS vs. HS	CS vs. HS	RM vs. HM	CM vs. HM
Metabolic pathways	4 (18,227)	2 (59,265)	1 (104,614)	3 (33,689)
RNA transport	3 (8970)	2 (46,110)	1 (132,455)	4 (2561)
Biosynthesis of secondary metabolites	4 (−14,014)	3 (37,358)	2 (86,457)	1 (195,966)
Fatty acid biosynthesis	4 (−1351)	1 (8954)	2 (263)	3 (−489)
Photosynthesis	1 (7784)	3 (670)	2 (873)	4 (85)
Photosynthesis—antenna proteins	1 (45,663)	3 (617)	2 (749)	4 (−1087)
Carbon fixation in photosynthetic organisms	1 (12,260)	2 (3551)	3 (2307)	4 (102)
Ribosome	3 (6016)	1 (9419)	4 (4067)	2 (8488)
Carbon metabolism	1 (15,852)	3 (7677)	2 (15,470)	4 (1305)
Plant hormone signal transduction	4 (−4483)	2 (2384)	3 (−533)	1 (5448)
Biosynthesis of amino acids	2 (9042)	3 (9019)	1 (11,309)	4 (912)
Flavonoid biosynthesis	(1338)	(1264)	(1677)	(1357)
Isoflavonoid biosynthesis	(−725)	(−18)	(1617)	(532)
Protein processing in endoplasmic reticulum	(−15,263)	(−3309)	(124,955)	(−64,763)

## Supplementary Materials

The following are available online at https://www.mdpi.com/2223-7747/9/9/1141/s1, Figure S1: GO analysis of the upregulated and downregulated genes between the *Brassica* hexaploid and its parents at two stages; Table S1: A list of the 3166 differentially expressed genes between the *Brassica* hexaploid and its parents at the full-size stage; Table S2: A list of the 3893 differentially expressed genes between the *Brassica* hexaploid and its parents at the mature stage; Table S3: A list of 10,564 differentially expressed genes between two developing stages in the *Brassica* hexaploid and its parents; Table S4: The considerably changed KEGG pathways between the two stages in the *Brassica* hexaploid and its parents; Table S5: A list of the non-additive genes of the *Brassica* hexaploid compared to its parents; Table S6: A list of putative TF genes of the DEGs among the *Brassica* hexaploid and its parents at two stages; Table S7: Primers used in the qRT-PCR analysis of gene expression in the *Brassica* hexaploid and its parents, and *ACT2/7* is used as the internal control to standardize the results. All raw RNA-seq data were submitted to the Sequence Read Archive (https://trace.ncbi.nlm.nih.gov/Traces/sra/) in NCBI under the BioProject ID code PRJNA482739, and accession number SRR7589740 to SRR7589757.
