# Supplementary material for: Comprehensive Transcriptomic Analysis for Developing Seeds of a Synthetic Brassica Hexaploid
Source: Plants (Basel). 2020 Sep 3;9(9):1141. doi: 10.3390/plants9091141 (PMC7570109; doi:10.3390/plants9091141)
Supplement: Supplementary file 1 [file plants-09-01141-s001.zip › Supplementary File/Figure S1.docx]

**Supplementary Materials:**


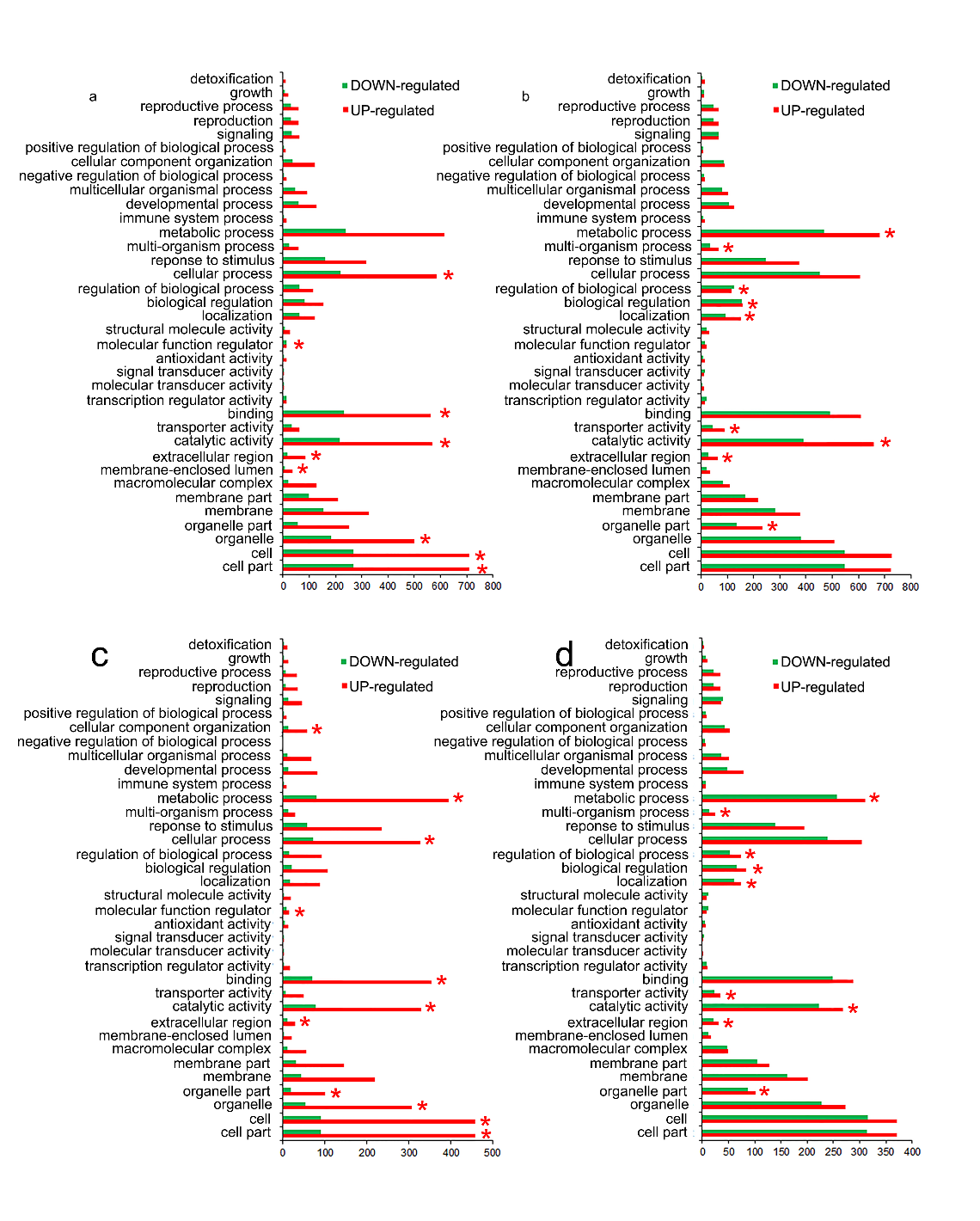


**Figure S1 GO analysis of up-regulated and down-regulated between *Brassica* hexapolid and its parents at two stages.**

**a** GO analysis of up-regulated and down-regulated genes in RS-VS-HS. **b** GO analysis of up-regulated and down-regulated genes in RM-VS-HM. **c** GO analysis of up-regulated and down-regulated genes in CS-VS-HS. **d** GO analysis of up-regulated and down-regulated genes in CM-VS-HM. RS, *B. rapa* at full-size stage; RM, *B. rapa* at mature stage; HS, *Brassica* hexaploid at full-size stage, HM, *Brassica* hexaploid at mature stage; CS, *B. carinata* at full-size stage; CM, *B. carinata* at mature stage.
