# Supplementary material for: Comprehensive Transcriptomic Analysis for Developing Seeds of a Synthetic Brassica Hexaploid
Source: Plants (Basel). 2020 Sep 3;9(9):1141. doi: 10.3390/plants9091141 (PMC7570109; doi:10.3390/plants9091141)
Supplement: Supplementary file 1 [file plants-09-01141-s001.zip › Supplementary File/Table S4.docx]

**Table S4 The considerably changed KEGG pathways between two stages in *Brassica* hexaploid and its parents.**

The top 10 of up-regulated pathways and down-regulated pathways based on FPKM in *Brassica* hexaploid and its parents at mature stage compared to the full-size stage. RS, *B. rapa* at full-size stage; RM, *B. rapa* at mature stage; HS, *Brassica* hexaploid at full-size stage, HM, *Brassica* hexaploid at mature stage; CS, *B. carinata* at full-size stage; CM, *B. carinata* at mature stage.

|  | Top 10 of  up-regulated pathways (RS-RM) | Top 10 of down-regulated pathways (RS-RM) | Top 10 of up-regulated pathways (HS-HM) | Top 10 of down-regulated pathways (HS-HM) | Top 10 of up-regulated pathways (CS-CM) | Top 10 of down-regulated pathways (CS-CM) |
| --- | --- | --- | --- | --- | --- | --- |
| 1 | Peroxisome  (88792) | RNA transport  (-3513388) | Other glycan degradation  (413844) | RNA transport  (-1611240) | Other glycan degradation  (557581) | RNA transport  (-706219) |
| 2 | Glycosphingolipid biosynthesis - globo and isoglobo series  (60348） | Protein export  (-1728228) | Biosynthesis of secondary metabolites (292590) | Protein export  (-768077) | Biosynthesis of secondary metabolites  (426845) | Protein export  (-333000) |
| 3 | Phosphatidylinositol signaling system  (49736) | Endocytosis  (-1718274) | Spliceosome  (260744) | Pyrimidine metabolism  (-755859) | Metabolic pathways  (361621) | Pyrimidine metabolism  (-309773) |
| 4 | Arginine and proline metabolism  (47762) | Pyrimidine metabolism  (-1718098) | Peroxisome  (258952) | Ubiquitin mediated proteolysis  (-748178) | Peroxisome  (342188) | Ubiquitin mediated proteolysis  (-300247) |
| 5 | Sphingolipid metabolism  (42562) | Ubiquitin mediated proteolysis  (-1705691) | Metabolic pathways  (284990) | Endocytosis  (-668994) | Spliceosome  (311129) | Selenocompound metabolism  (-286262) |
| 6 | Galactose metabolism  (40293) | Selenocompound metabolism  (-1662812) | Starch and sucrose metabolism (148903) | Selenocompound metabolism  (-668897) | Galactose metabolism  (192848) | Endocytosis  (-131004) |
| 7 | Glutathione metabolism  (38404) | Protein processing in endoplasmic reticulum  (-1555259) | Glyoxylate and dicarboxylate metabolism  (139908) | Protein processing in endoplasmic reticulum  (-483603) | Glyoxylate and dicarboxylate metabolism  (173795) | Photosynthesis - antenna proteins  (-100813) |
| 8 | MAPK signaling pathway - plant  (21482) | Metabolic pathways  (-580090) | Galactose metabolism  (131399) | Photosynthesis - antenna proteins  (-65343) | Glycerolipid metabolism  (126007) | Photosynthesis  (-68817) |
| 9 | Spliceosome  (17464) | Biosynthesis of secondary metabolites  (-467025) | Cyanoamino acid metabolism (126881) | Biosynthesis of amino acids  (-60690) | Biosynthesis of unsaturated fatty acids  (124381) | Carbon fixation in photosynthetic organisms  (-45479) |
| 10 | mRNA surveillance pathway  (13307) | Cutin, suberine and wax biosynthesis  (-166545) | Phosphatidylinositol signaling system  (89607) | Phagosome  (-37897) | Fatty acid metabolism  (120976) | Biosynthesis of amino acids  (-45151) |
