# Supplementary material for: Comprehensive Transcriptomic Analysis for Developing Seeds of a Synthetic Brassica Hexaploid
Source: Plants (Basel). 2020 Sep 3;9(9):1141. doi: 10.3390/plants9091141 (PMC7570109; doi:10.3390/plants9091141)
Supplement: Supplementary file 1 [file plants-09-01141-s001.zip › Supplementary File/Table S7.docx]

| **Gene ID** | **Sense primer** | **Anti-sense primer** |
| --- | --- | --- |
| ACT2/7 | TTCAATGTCCCTGCCATGTA | GAGACGGAGGATAGCGTGAG |
| Bra005287 | CAGACAACAGCAACGGACG | TCTAACTACACCAGCCTTCACC |
| Bra013832 | GACGCACTAACCTCCCTTTC | CGCTCACATCTCCGCTTTC |
| Bra007100 | ACCGCCTGAGCCTCTATTC | TCTGGCTCTTGTAACCAATGAC |
| Bra027057 | AGGACTCGTTCAGGAGGTTG | GTTGGTGGGCTACTGTTATTCA |
| Bra001257 | GCGTATGCTTGGGGTGAGA | GGCTGGTGAGGTAAAGGGAG |
| Bra008589 | CAAGGTGGCGAGCATAGTG | CAGGTGGGGTGTAGTTAGGAG |
| Bra010283 | CCTTGGATTCTCAGGCTATGT | TCTGGTCCCTTCCTTGCTC |
| Bra013872 | TACGACGGAGGTGAAAATGA | GTTTAGCACCTGGCTGATACTC |
| Bra020639 | AATACGCAGTAACAGTGGAAAGC | TGGGTGACGATACAGTGGC |
| Bra019774 | GAGGAGGAGATGGTGAAGTGTC | CTATCGCAACCTCGGCAAC |

**Table S7 Primers used for amplification in qRT-PCR**
